# Old Folks, Bad Boon: Antimicrobial Resistance in the Infant Gut Microbiome

**DOI:** 10.3390/microorganisms11081907

**Published:** 2023-07-27

**Authors:** Silvia Saturio, Alejandra Rey, Anna Samarra, Maria Carmen Collado, Marta Suárez, Laura Mantecón, Gonzalo Solís, Miguel Gueimonde, Silvia Arboleya

**Affiliations:** 1 Instituto de Productos Lácteos de Asturias (IPLA-CSIC), 33300 Villaviciosa, Spain; silvia.saturio@ipla.csic.es (S.S.); a.rey.marino@ipla.csic.es (A.R.); 2 Instituto de Investigación Sanitaria del Principado de Asturias (ISPA), 33011 Oviedo, Spain; msr1070@hotmail.com (M.S.); laura_mantecon@hotmail.com (L.M.); gsolis@telefonica.net (G.S.); 3 Institute of Agrochemistry and Food Technology (IATA-CSIC), 46980 Paterna, Spain; asammas@iata.csic.es (A.S.); mcolam@iata.csic.es (M.C.C.); 4 Pediatrics Service, Central University Hospital of Asturias (HUCA-SESPA), 33011 Oviedo, Spain

**Keywords:** intestinal microbiome, antibiotic resistance genes, infant, resistome, probiotics

## Abstract

The development of the intestinal microbiome in the neonate starts, mainly, at birth, when the infant receives its founding microbial inoculum from the mother. This microbiome contains genes conferring resistance to antibiotics since these are found in some of the microorganisms present in the intestine. Similarly to microbiota composition, the possession of antibiotic resistance genes is affected by different perinatal factors. Moreover, antibiotics are the most used drugs in early life, and the use of antibiotics in pediatrics covers a wide variety of possibilities and treatment options. The disruption in the early microbiota caused by antibiotics may be of great relevance, not just because it may limit colonization by beneficial microorganisms and increase that of potential pathogens, but also because it may increase the levels of antibiotic resistance genes. The increase in antibiotic-resistant microorganisms is one of the major public health threats that humanity has to face and, therefore, understanding the factors that determine the development of the resistome in early life is of relevance. Recent advancements in sequencing technologies have enabled the study of the microbiota and the resistome at unprecedent levels. These aspects are discussed in this review as well as some potential interventions aimed at reducing the possession of resistance genes.

## 1. Introduction

The establishment of the gut microbiota is a process that starts mainly after birth and is affected by several perinatal factors [1]. Although the process of defining a healthy infant microbiota is still ongoing, we assume that the composition of the gut microbiota of infants that are born full-term, vaginally delivered, without having contact with antibiotics (both in the cases of their mothers and themselves), and exclusively fed their own mother’s milk, is the “golden” standard. The correct ensemble of this microbial conglomerate entails the possession of a healthy immunological and physiological status with long-lasting effects [2]. However, even in this hypothetical ideal context, the gut microbiota foundation also involves the natural establishment of a set of antimicrobial resistance genes (ARGs) present in different populations of microorganisms, known as the gut resistome [3]. 

Antibiotics are the drugs that are the most administered during early life, understood as the prenatal to postnatal period. During pregnancy, it was estimated that one out of five European women is prescribed at least one antibiotic, but the rate is double in the United States [4]. More than 70% of newborns admitted to neonatal intensive care units (NICUs) receive antibiotics [5], and children as young as one year of age [6] are prescribed the most antibiotics; prevalence is particularly high in children younger than six years of age in Europe [7]. The use of antibiotics saves lives, but not without challenges. The administration of antibiotics early in life can contribute to microbiota–host crosstalk disruption with short- and long-lasting effects, contributing to a greater risk of disease both in childhood and in adulthood [8]. Moreover, there is increasing evidence showing that antimicrobial resistance (AMR) is a significant issue in NICUs across the world [9], and it is estimated that it might be a considerable problem in pediatric care sooner rather than later in the first half of this century. 

At present, AMR is 1 of the top 10 global public health threats that humanity faces [10]. It occurs when bacteria, viruses, fungi, or parasites change over time following a natural evolutionary process and acquire new characteristics that reduce or stop their susceptibility to antimicrobials, making infections harder to treat and increasing the risk of disease spread, severe illness, and death [10]. An estimated four million human deaths worldwide were linked to antimicrobial resistance in 2019, including 1.3 million human deaths directly caused by resistant bacteria [11]. The European Centre for Disease Prevention and Control estimated that, each year, more than 670,000 infections occur in the European Union and European Economic Area (EU/EEA) due to bacteria resistant to antibiotics and that approximately 33,000 people die as a direct consequence of these infections [12]. The costs of antimicrobial resistance to national economies and their health systems are also significant. The Organization for Economic Cooperation and Development estimates that the associated costs to the health systems of EU/EEA countries are approximately EUR 1.1 billion per year [12], with these costs to the global economy estimated to be a loss of USD 100 trillion by 2050 [13].

The causes of AMR are complex; it can arise as a natural phenomenon. Evolution via natural selection can involve the occurrence of antibiotic resistance and ARGs from non-clinical environments may be transferred from non-pathogenic bacteria to other potential pathogens. Pioneer studies unveiled the presence of ARGs in remote communities with minimal antibiotic exposure [14], suggesting complex mechanisms of AMR development, and the importance of environmental bacteria as a source of ARGs. However, the main drivers of AMR are believed to have been induced by the misuse, overuse, and short-cut prescription use of antimicrobials in humans, animals, and agriculture [15]. As an example, in data from the EU/EEA in 2020, both the community and hospital consumption of broad-spectrum antibiotics was 3.5 times higher than the administration of narrow-spectrum antibiotics, which should normally be the first-line therapy [12]. A study providing longitudinal estimates for human antibiotic consumption covering 204 countries and 19 years (2000–2018) highlighted the increment in the global antibiotic consumption rate of 46 percent in the last two decades [16]. The wide use of antibiotics provides selection pressure, causing an increment in bacteria that contain ARGs, and the spread of AMR could make many pathogens much more lethal. Moreover, since antibiotic-resistant bacteria do not comply to borders, usage in neighboring countries may increase resistance in others, independent of the use in that specific country, due to the international mobility of people, animals, and goods. It is worth pointing out that it was recently estimated that the prevalence of resistant bacteria increases immediately after usage and continues to increase for at least 4 years after antibiotic usage [17].

The gut resistome has to be understood from the One-Health (human–animal–environment) perspective as the collection of all types of ARGs (acquired and intrinsic resistance genes), their precursors (including pathogens, antibiotic producers, and non-pathogenic microorganisms found either free living in the environment or as commensals with other organisms), and some potential resistance mechanisms within microbial communities that require evolution or alterations in the expression context to confer resistance [18]. Recently, the development of novel and powerful techniques for the assessment of the intestinal microbiome has increased our knowledge of the role of the microbiome as an ARG reservoir. The gut resistome development starts with life and, as it happens with the microbiome, several pre-, peri-, and postnatal factors are involved in its modulation. Therefore, understanding the impact of these factors is critical in the fight against AMR. In this review, we aim to discuss the current knowledge concerning the development of the gut resistome in the infant. Moreover, potential intervention strategies targeted at reducing the ARGs load are considered as well.

## 2. The Study of Antimicrobial Resistance in the Human Microbiome

Classically, the study of the gut microbiota has been conducted via quantitative culture-dependent techniques that use different media to select specific groups of microorganisms based on their metabolic requirements in a simple, cost-effective, and easy way [19]. However, these methods have certain limitations since the selected culture media are often unable to distinguish between closely related phylogenetic groups, thus requiring the use of numerous morphological, physiological, and metabolic tests to discriminate amongst them. Due to the laboriousness of these techniques, the number of samples that can be processed is reduced. In addition, most gut bacteria are anaerobic, which makes it difficult to establish the optimal conditions for their culture. Since these methods are insufficient to characterize the complexity of the gut microbiota, a wide variety of molecular techniques are used at present. 

PCR allows the amplification of specific regions of DNA and RNA, and multiple variants of this procedure are used. Microarrays are a high-throughput molecular screening technique that can also be used to detect the presence of certain gut bacteria by the simultaneous hybridization of several probes on the same substrate [20]. Generally, these methods are used along with culture-dependent techniques in the clinical setting. Recent advancements in sequencing technologies have resulted in a reduction in their cost and an expansion of their use for the study of the microbiota. Metagenomics enable the characterization of the gut microbiota by isolating DNA directly from the sample without the need for culture or a priori knowledge of the genes of interest. Other high-throughput technologies have been developed and applied to measure multiple omics data types, such as transcriptomics, culturomics, and metabolomics, which, used in combination, have the potential to unravel new molecular mechanisms of the interaction between the members of the gut microbial community and their niche [21]. 

### 2.1. Omics Techniques for Monitoring ARG in the Microbiome

Since the first culture-based study conducted in 1970 by Finegold and collaborators with the objective of examining the effects of various antimicrobial compounds on the composition of the intestinal microbiota [22], many techniques have been developed for this purpose. However, most of them only enable the detection of a few well-studied resistance genes conducted with known and cultivable bacteria, so their use for a broad-spectrum screening in non-cultivable bacteria was not possible. The subsequent development of molecular techniques, such as PCR assays, enabled the detection of antimicrobial resistance genes and possible mutations [23], although these methods are not well suited for the identification and quantification of these genes in complex microbial communities. The use of real-time qPCR has enabled the identification and quantification of antibiotic resistance genes in the oral and fecal samples of neonates [24,25]. Although this technique has considerable value, since it allows the absolute quantification of known ARGs, it could not be used for a wide-spectrum screening. 

The development of high-throughput sequencing technologies has, therefore, made it possible to examine the genetic information of intestinal bacteria in depth, thus allowing the detection of new antibiotic resistance genes. In the last decade, several studies have been conducted in which different approaches have been used, allowing the quantification of hundreds of antibiotic resistance genes [26]. Functional metagenomics characterize and identify novel resistance genes by the selection of shotgun-cloned DNA fragments that can confer survival to an indicator host, such as *Escherichia coli* [27]. Other approaches based solely on high-throughput sequencing techniques are targeted gene sequencing or amplicon sequencing, which have been used to identify mutations or variants in antimicrobial resistance genes and their genetic context [28,29], and shotgun metagenomics, which is able to analyze the global resistome of the sample [30]. Information obtained through metagenomics combined with other omics approaches, such as transcriptomics, metabolomics, proteomics, or culturomics, can lead to the discovery of novel ARGs and the molecular mechanisms that confer AMR to pathogenic bacteria. Transcriptomics, for example, made it possible to fill the gap between the resistance phenotype and the genes involved [31], and the role of non-coding regulatory RNAs in the resistance phenotype was identified [32]. These RNAs are able to modulate the expression of ARGs in different microorganisms, as in the case of the transcriptional attenuation of the *tet*M gene from *Enterococcus faecium* by the small RNA molecule *Ern0030* [33].

### 2.2. Challenges in Identifying ARGs in the Microbiome

Despite representing a breakthrough in the discovery of ARGs and the molecular mechanisms underlying resistance, high-throughput sequencing technologies have some drawbacks when it comes to performing this task. First, the assembly of short reads generated by technologies, such as Illumina, is computationally expensive, and their subsequent annotation depends on the quality of the reference databases, which often lack efficient and sustainable curation pipelines, leading to conflicting gene names and redundancy across databases. For this reason, it is important to create standardization protocols for the biocuration of reads from sequencing technologies that allow the identification and characterization of protein-coding resistance genes and other non-coding genes that may be implicated in AMR mechanisms [34]. The recent development of the long-read sequencing techniques simplify and improve genome assembly.

Machine learning algorithms, which build a predictive model that can be applied to query sequences to predict their outcome, were used by numerous studies to predict the resistance phenotype from the genotype directly, but the accuracy level was too low to be able to apply this approach for clinical diagnostics [35]. This is due to the high dependence on the training data set and a priori knowledge; in the future, large, curated data sets of antibiotic resistance genes are required to develop robust models that improve the accuracy of the predictions. A tool named *NanoARG* that uses deep learning to identify resistance genes has recently been developed [36]. This approach, which uses one model for assembled genes and another for short reads, has high precision, reducing false negatives in the metagenomic discovery of ARGs. 

In summary, although the identification of ARGs in complex ecosystems has always been challenging, the current availability of sequencing techniques has enabled unprecedented high-throughput screening for the presence of such genes in the human microbiome.

## 3. The Infant Intestinal Resistome

The gut microbiota undergoes major changes early in life, until it begins to stabilize during childhood [1]. The first days of life are characterized by the presence of species with a facultative aerobic metabolism, mainly belonging to the *Enterobacteriaceae* family, which oxidize the intestinal environment favoring the emergence of strict anaerobes, and bifidobacteria stand out as one of the dominant groups during the first year of life. Once solid foods are introduced into the diet, microbial diversity increases [37]. In this microbial colonization context, the gut resistome is established in parallel with microbial communities; therefore, the gut becomes a reservoir of ARGs [38]. Although the composition of the neonatal gut microbiota is well characterized at the taxonomic level, studies of the development of the resistome are still scarce.

A recent systematic review on the neonatal intestinal resistome [39] observed a higher presence of ARGs in newborns when compared to their mothers’ microbiota. Gene transfer from mother to child occurs, considering that even newborns who have not been exposed to antibiotics harbor a considerable load of ARGs. During the first months of life, resistance genes to aminoglycosides, beta-lactams, erythromycin, and other antibiotics are commonly found in the neonatal gut, with efflux pumps conferring resistance to different types of antibiotics being the most common of all [40]. It has been postulated that these high loads of ARGs are mainly due to the presence of specific bacterial groups, such as *E. coli* and various species of the *Staphylococcus* genus [40]. More specifically, *erm*B, *erm*C, *mef*A (which confer resistance to macrolides) *tet*A, *tet*B (which confer resistance to tetracyclines), *mec*A, *bla*SHV, and *bla*CTXM-1 (which confer resistance to beta-lactams) were detected in the gut microbiota of three-day-old infants. Of these, *mef*A, *erm*B, and *erm*C were the most widely distributed. In the same study, mother–neonate pairs were also analyzed separately, showing that only two of the genes (*erm*B and *mef*A) were shared in all cases; there were cases in which the newborns had ARGs that their mothers did not, and vice versa. This suggests that the presence of ARGs in the neonatal gut microbiota is not only due to the transfer of microorganisms from the maternal microbiota but also from other sources, such as the hospital environment [25]. Later on, with infant growth, the microbial diversity of the neonatal microbiota changes, also affecting the resistome. The metagenomic analysis of more than 662 fecal samples from one-year-old children [41] found 409 types of antibiotic resistance genes, of which 40.8% conferred resistance to multiple antibiotics. Although neither tetracyclines nor fluoroquinolones are commonly used antibiotics in pediatric patients and no antibiotics were administered to any of the children in the cohort, genes conferring resistance to them were the most commonly detected. In the same study, they concluded that samples with similar bacterial profiles also had similar resistomes.

**Figure 1:**
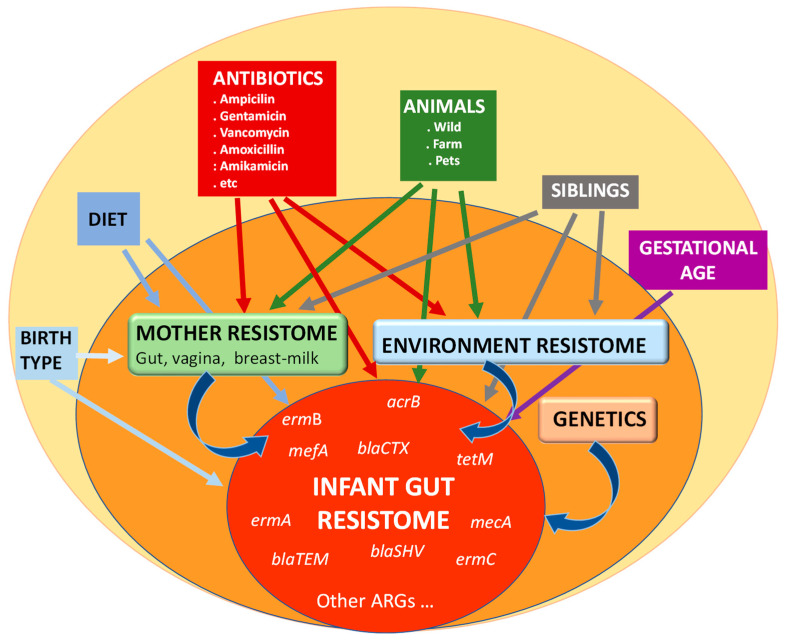
Main factors influencing the establishment and development of the resistome in the infant gut.

From birth, and even before it, several factors are present that may affect the establishment and later development of the gut microbiota and, therefore, that of the resistome (Figure 1). Several studies have demonstrated the importance of gestational age, birth type, environment, and diet as modulators of the gut microbiota in the early stages of life [1]. Of course, antibiotics also play a crucial role, either when administered to the mother during pregnancy or delivery, which alters the vaginal microbiota and prevents the transfer of species considered beneficial, or when administered directly to the newborn [42,43]. It is also important to consider that, during the first months of life, *Enterobacteriaceae*, a microbial family often associated with the presence of ARGs in the gut [44], is among the dominant microorganisms in the intestinal microbiota, which is dominated by the phyla Actinomycetota and Pseudomonadota in contrast to the adult microbiota dominated by Bacillota and Bacteroidota. This is likely to contribute to the higher levels of ARGs in infants’ microbiota when compared to their mothers [45].

Among the different factors known to affect the newborn microbiota development, and thus potentially affecting the resistome, the impact of gestational age and mode of delivery are well known. Gestational age is one of the most studied perinatal factors that directly affects the gut microbiota. Children born prematurely have a more immature immune and gastrointestinal systems. As a result, they tend to be admitted to hospital for longer periods of time and receive medication more frequently than full-term infants. According to data collected in a recent review [46], differences could be found in the composition of the meconium, while the Bacillota phylum was dominant in both groups of infants. The authors found that, in full-term infants, the presence of *Bacillus* species was higher, and in pre-term infants, *Enterococcus* and *Staphylococcus* were dominant. Likewise, in the pre-term group, after a few days of life, members of the phylum Firmicutes decrease, and those of the phylum Pseudomonadota, such as the families *Pseudomonadaceae*, *Enterobacteriaceae*, and *Vibrionaceae*, increase, which may contribute to the higher levels of ARGs in these infants. Prolonged stays in NICUs mean that pre-term infants, unlike those born at full term, have a higher proportion of strains found in the hospital environment in their microbiota, such as *E. coli*, *Klebsiella* spp., *Staphylococcus* spp., and *Enterococcus* spp., many of which are related to nosocomial infections and show resistance to multiple antibiotics [40]. In relation to the types of ARGs found in pre-term infants, there has been a dominance of genes conferring resistance to beta-lactams, amphenicols, polymyxins, and tetracyclines, even though the latter is not used as an antibiotic of choice in NICUs [47]. 

Depending on the type of delivery, significant differences have also been found among the microbial populations present in the gut microbiota. During a vaginal birth, the newborn passes through the birth canal, with the bacteria present in the mother's vagina acting as the initial inoculum. They thus present an intestinal microbiota dominated by *Prevotella* spp. and *Lactobacillus*, with the presence of bifidobacteria increasing with the passage of days. In contrast, in those born by caesarean section, whose first contact with the outside world is with the hospital environment and the mother's skin, we find a microbiota dominated by *Corynebacterium*, *Staphylococcus*, and *Cutibacterium* spp. [48]. At one month of age, vaginally delivered infants had a higher abundance of Bacteroides and Parabacteroides [49]. Additionally, caesarean deliveries have been associated with high burdens of ARGs in the neonatal gut microbiota during the first month of life, with genes conferring resistance to glycopeptides, phenicol, and sulfonamide being the most prevalent [40].

## 4. The Origin of Infant Resistome: Vertical Transmission of ARGs

The infant receives its primary microbial inoculum from the mother, and it is later strongly affected by the maternal–filial interaction, which drives the vertical transmission of microbes and the ARGs they contain. Therefore, the resistome of pregnant women, and that of breast milk in the case of breast-fed babies, is of prime importance.

### 4.1. Pregnancy Microbiota Changes

During pregnancy, several physiological adaptations occur, and the women’s microbiota is also subject to these modifications. Whereas the gut microbiota in the first trimester of pregnancy is very similar to the microbiota of non-pregnant women, in the second trimester, the microbiota suffers an increase in lactic acid bacteria and a reduction in butyrate-producing bacteria [50]. During the third trimester of pregnancy, the butyrate-producing bacteria decline, whereas *Proteobacteriaceae*, *Bifidobacteriaceae*, and lactic acid bacteria increase [50,51]. The bacterial groups Gammaproteobacteria, in the Pseudomonadota phylum, are among the major carriers of ARGs, while Bacillota is also enriched in pregnant women and can carry ARGs as well [52]. Indeed, correlations between the phylum Pseudomonadota and ARG/mobile genetic elements have been reported [53].

In the vagina, as well as in the endometrium, *Lactobacillus* represents the most dominant genus of pregnant women [54], together with anaerobic microorganisms, such as *Prevotella*, *Garnerella*, *Finegoldia*, and *Dialister*. During pregnancy, a reduction in the diversity of pregnant women’s vaginal microbiome and a higher abundance of Clostridiales, Bacteroidales, and Actinomycetales has been described [50]. As expected, the presence of ARGs is positively correlated with *Dialister*, *Atopobium*, *Prevotella, Gardenella*, and *Anaerococcus*, and negatively related to the abundance *Lactobacillus* spp. [55] in the vagina. Cabralla-Maestre and co-workers [56] reported that tetracycline resistance genes were the most frequent ARGs in the vagina of healthy non-pregnant women, followed by multidrug resistance genes. In comparison with the gut resistome, the vagina has a lower diversity in terms of antibiotic resistance [54,57]. A complete knowledge of the vaginal resistome of pregnant women and how it is modulated is crucial as it has a direct impact on the infant’s early acquisition of resistance through direct contact during vaginal delivery. 

It is known that the oral cavity is also a reservoir of highly diverse and rich microbiota, and it has been widely characterized [58]. According to research conducted on Japanese women, there was a higher presence of *Porphyromonas gingivalis* and *Aggregatibacter actinomycetemcomitans* during the early and middle stages of pregnancy as compared to non-pregnant individuals. The *Candida* species were found to be more abundant during the middle and late stages of pregnancy [59,60]. However, there is relatively little literature indicating how pregnancy changes in the oral microbiome are induced and the development of the oral resistome during pregnancy and the influence on the offspring.

### 4.2. ARGs in Pregnancy and Lactation

The modulation of the maternal microbiome has emerged as an important factor in gestational health and outcomes and is associated with the later establishment of the infant’s own microbiota. In addition, these changes are also directly related to the establishment of AMRs during pregnancy and their later transmission to the offspring, but there is little evidence of this. These shifts in the maternal microbiota are due to the biological process of pregnancy, as previously discussed, but may also be affected by external factors to which the woman is exposed during that period of time [61]. A study conducted by Serrano and collaborators [62] found that the resistome varied depending on the gestational age and the mode of delivery. In addition, other external factors, such as antibiotic administration and diet, influence the gut microbiome and resistome during pregnancy [49]. However, other studies reported that the antibiotic resistance harbored by the gut microbiota of pregnant mothers is not related to age, BMI, or pregnancy status [53]. Overall, the evidence suggests that more effort is needed to describe the changes that modulate the mother’s resistome during pregnancy and the role it may have on the establishment of the offspring’s resistome.

### 4.3. The Maternal–Filial Transfer Route as a Source of ARGs for the Neonate

The colonization of newborns by antibiotic-resistant microorganisms occurs at the moment of birth or soon after through the contact with the mothers’ uterus, skin, or the environment of the hospital. For this reason, ARGs have been identified in newborns within hours of birth [63]. Even though the prevalence and the specificity of resistant strains may vary between mothers and infants, recent research provided evidence on the vertical transmission of ARGs from mothers to offspring [43,64]. Highly identical antibiotic-resistant *Limosilactobacillus fermentum*, *Lactobacillus gasseri*, *Bifidobacterium longum*, and *Enterococcus faecalis* have been isolated in both mothers and infants [65], which supports the theory of vertical transmission during the early stages of life. Different studies have indicated that antibiotic-resistant microbes can be present in a mother’s vaginal tract and subsequently passed on to her offspring [66,67]. For example, strains of *Prevotella*, *Lactobacillus*, *Ureaplasma*, *Gardnerella*, *Corynebacterium*, and *Staphylococcus* resistant to tetracycline have been found in infants’ feces as a result of vertical transmission from the mother [68]. Tetracycline resistance is the most common in the adult gut microbiota, carrying *tet*M, *tet*O, and *tet*W genes [55,69]. 

Breastfeeding provides numerous benefits for infants, including the transfer of maternal immune cells and antibodies as well as vital nutrients and bacteria [70]. Human milk also serves as a consistent source of commensal and potentially probiotic bacteria for the newborn’s gut, with *Lactobacillus*, *Bifidobacterium*, *Staphylococcus*, and *Streptococcus* being the most dominant genera in a woman's milk [71]. Nevertheless, breastmilk plays a crucial role in the acquisition of antibiotic resistance in early life. Human milk has been found to contain antibiotic-resistant bacteria, such as *Staphylococcus*, *Streptococcus*, *Acinetobacter*, *Enterococcus*, and *Corynebacterium* resistant to antibiotics, and multidrug-resistant profiles have been isolated in human milk [45,70,71]. Antibiotic-resistant bacteria were observed in breastmilk as a potential source of ARGs in infants, so that infants shared gut resistomes and mobilomes with their own mother’s breastmilk microbiota [54]. A study reported that 70% of the ARGs detected in breastmilk are present in infant feces, and similar patterns were observed in the mobilome [72]. Moreover, metagenomic analyses have highlighted the high levels of ARGs and mobile genetic elements in breastmilk and the similarity of breastmilk and the infant’s gut resistome [72]. The transmission of AMR through breastmilk has also been proved with the detection of multi-drug-resistant strains of *Streptococcus* and *Staphylococcus* in both breastmilk and infant fecal samples [73,74]. Similarly to gut microbiota, antibiotic-resistant microorganisms found in human milk have been related to the administration of certain antibiotics to mothers during lactation [75].

Therefore, a mother can impact the infant's gut resistome by transmitting resistant bacteria, although ARGs can also come from other parts of her body or external sources. The elevated use of antibiotics during pregnancy and breastfeeding constitutes a risk factor for the development of AMR in the mother's gut and breastmilk resistome, which can then be passed on to the offspring [66]. Overall, more knowledge about the mother’s gut and breastmilk may be important to decipher how neonates acquire antibiotic-resistant genes in early life and the role that environmental factors may play, so that new approaches and decisions can be made to reduce AMR.

## 5. Impact of Antibiotics on the Developing Microbiota Composition

### 5.1. Antibiotics Use in Early Life

The use of antibiotics in pediatrics covers a wide variety of possibilities due to the different ages and diagnostic conditions that can arise, and it is one of the most widely used drug classes from birth to the end of pediatric age [76]. Their high consumption presents important geographical differences, with a clear north–south gradient in Europe, but also according to other factors, such as the type of prescriber or the responsible healthcare system [77,78]. In recent years, the publication of official guidelines on antibiotic usage improved prescription practice and exposure in the pediatric population [79]. It is important to point out, however, that the monitoring systems based on defined daily doses (DDDs) [80] were designed not for children but for adults, and that children have peculiarities of dosage by weight and need special pharmaceutical presentations (syrups and drops). For these reasons, they are a population in which the consumption of antibiotics is difficult to measure and control [77].

The use of antimicrobials in each health setting (hospital or extra-hospital) and age group (neonates, infants, and older children) depends on the most frequent causative microorganisms, and their choice will be empirical until culture results and antibiograms are available. The characteristics of the patient (age, previous pathology, and clinical situation) will be essential for the selection of the drug and administration regimen. In any case, the antibiotics used and the guidelines and duration of treatment are under continuous discussion [81].

#### 5.1.1. Use of Antibiotics in Neonatology

Classically, neonatal infections are divided into early and late infections (Table 1), although it is currently debated whether it is better to use the terms vertical (mostly early) and nosocomial (mostly late) infections. On the other hand, the definitions of neonatal sepsis, its diagnostic methodology, and, therefore, its treatment are currently changing with the use of clinical and biochemical markers, as well as new microbiological techniques [82,83]. Early vertical infections have their origin in the mother, via the ascending route but also the transplacental route. Therefore, they are usually caused by bacteria that colonize the maternal genitourinary tract and/or by microorganisms that cross the blood–placental barrier due to a generalized maternal infection. Therefore, treatment must cover both Gram-positive (*Streptococcus agalactiae*, *Listeria*, or *Enterococcus*) and Gram-negative microbes (especially *E. coli*). For this reason, the most frequent combination is based on the use of an ampicillin-type beta-lactam and a gentamicin-type aminoglycoside [84,85].

Nosocomial infections originate in neonatal intensive care units (NICUs), where risk factors, such as immune immaturity, routes of infection (central catheters and mechanical ventilation), and long admission times and treatments (antacids, intravenous feedings, previous antibiotic therapies, and surgeries), are often present. These nosocomial infections are usually caused by Gram-positive organisms, such as *Staphylococcus epidermidis* or *S. aureus*, or by Gram-negative ones, such as *E. coli*, *Klebsiella pneumoniae*, *Pseudomonas aeruginosa*, or *Serratia marcescens*. In order to cover both spectra, and until culture and antibiogram results are available, the combination of vancomycin or teicoplanin and a second-line aminoglycoside, the amikacin type, is the most used choice [84] (Table 1).

Neonatal infections of community origin, i.e., those developed at home, are usually of various origins: respiratory (of particular interest are *Chlamydia* infections, a vertical infection that would require the use of macrolides), soft tissue (omphalitis or mastitis, which require treatment with cloxacillin for *S. aureus*), or urinary (which would need empirical combinations, such as ampicillin plus gentamicin, to cover *Enterococcus* and *E. coli*, until culture results are available). Central nervous system infections, especially bacterial meningitis, require the use of cefotaxime, given its important passage through the blood–brain barrier and its usual coverage of the usual germs for this location [86].

#### 5.1.2. Use of Antibiotics in Infants and Older Children

Infants from 1 month to 2 years of age, vaccinated according to the routinely recommended guidelines, usually present infections of viral origin that do not require antibiotic therapy and, in most cases, are treated in the outpatient setting without the need for hospital admission. However, unnecessary antibiotics are administered more frequently in this age group due to initial diagnostic uncertainty and family anxiety. In children older than 24 months, the use of antibiotics is more directed and, with a few exceptions, it is possible to assess the choice of the drug with greater precision and make more appropriate use of them [79].

The most commonly used antibacterial prescriptions are penicillin for tonsillitis; amoxicillin at high doses in acute otitis media and sinusitis; and amoxicillin, amoxicillin–clavulanate, or macrolides in pneumonia and other bacterial respiratory infections of the lower respiratory tract [87,88] (Table 1). 

For urinary tract infections, the other large group of pediatric infections whose origin is usually bacterial (*E. coli*, *Proteus mirabilis*, and *Enterococcus*), amoxicillin–clavulanate or second- or third-generation cephalosporins (cefuroxime, cefotaxime, and ceftriaxone) are used, according to whether the cases are managed on an outpatient or hospital basis, respectively [89]. In the case of bone or joint infections, in which germs, such as *S. aureus* or *Kingella kingae*, usually play a leading role, the combination of cloxacillin plus cefotaxime in the hospital setting until cultures and antibiograms are obtained is usually the choice [90]. Meningeal infections and sepsis usually require the use of third-generation cephalosporins, associated or not with aminoglycosides (cefotaxime and gentamicin) until cultures are obtained [86,91] (Table 1). Other situations, such as infections in children with cancer or with significant chronic pathologies, are not addressed in this review since they do not represent common treatments.

All these different conditions and options may receive a large variety of doses and treatment regimens with the most frequently used antibiotics, both in neonates and older children, as summarized in Table 2.

### 5.2. Impact of Antibiotic Use on Microbiota Composition in Early Life

Disruption caused by antibiotics is especially important during the first months of life, as it increases the likelihood of the gut being colonized by potential and opportunistic pathogens and increasing the presence of ARGs [92]. For this reason, in recent years, there has been an increase in the number of studies focused on this aspect, although our knowledge is still scarce.

Before presenting the main results obtained to date, it is important to note that the effect that different antibiotics have on the bacterial populations present in the intestinal microbiota depends on several factors. These are very heterogeneous and include, as shown in the previous section, the type of antibiotic, the dose, the route of administration, and the duration of treatment. Moreover, the basal composition of the gut microbiota, which is also dependent on different factors, also determines the microbiota response to the antibiotic challenge. These are likely to explain the differences between the studies published in the literature. Most studies concluded that, following antibiotic treatment, there is a decrease in bacterial diversity in the infant gut, in particular in genera such as *Bifidobacterium*, *Lactobacillus*, and *Bacteroides* [93]. In addition to reduced diversity, the gut microbiota of antibiotic-treated infants is characterized by a reduced stability [94]. 

An analysis of the composition of the gut microbiota of newborns treated with ampicillin and gentamicin, one of the most commonly used combinations, showed that these infants had higher levels of the phylum Pseudomonadota and lower levels of Actinomycetota, in particular showing a decrease in bifidobacteria. The differences from the control group were mitigated over time, although two months after the finalization of the treatment, some differences, such as a reduced number of bifidobacterial species, were still observed [95]. In some studies, even after two years, the relative abundance of the *Bifidobacterium* genus was still lower than that in the control group [96]. Other authors also observed a decrease in the presence of bifidobacteria and other species belonging to the genera *Escherichia* and *Staphylococcus*, whereas the presence of the genera *Klebsiella* and *Enterococcus* increased. The impact of antibiotics on the microbiota composition seems to be mitigated over time; however, some differences are still apparent months after treatment [95,96,97]. In contrast, other authors reported no differences in microbiota composition between exposed and non-exposed infants at 1 year of age [41]. As mentioned at the beginning of this section, these differences could largely be due to the type of antibiotics/treatments used. For example, when comparing the effects of the combination of amoxicillin plus cefotaxime vs. penicillin plus gentamicin, it was observed that the latter treatment had less effect on the neonatal intestinal microbial communities [97]. It has also been shown in pre-term infants that some widely used antibiotics, such as penicillin, ampicillin, gentamicin, or vancomycin, favor the increment in enterobacteria, while decreasing the presence of bifidobacteria and members of the *Bacillaceae* and *Lactobacillaceae* families [98]. Time and treatment duration are other important aspects. During the first week of life, the prolonged I.V. administration of amoxicillin and ceftazidime to pre-term infants induced microbiota differences dependent on the exposure time. Both short and prolonged exposure reduced the levels of *Bifidobacterium*, but in infants subjected to the longer treatment, the levels did not recover afterwards [99]. A longer antibiotic exposure has also been associated with an increased risk of late-onset sepsis, necrotizing enterocolitis, and even death in pre-term infants [100]. In a study on infants treated with ampicillin and gentamicin, the authors found that a longer exposure increased the presence of enterobacteria, whereas a shorter treatment allowed the recovery of the initial state three weeks after treatment [101]. It has been shown that, as the time of exposure to antibiotics administered to infants in the NICU increased, the presence of anaerobic and butyrate-producing species decreased; the effect was greater in those treated with ampicillin and tobramycin [102]. 

Similar conclusions have also been reached by other authors who, after reviewing the literature, concluded that the microbiota of infants treated with antibiotics during the first year of life appears to recover over time. However, unlike infants not exposed to antibiotics, they have a higher proportion of multidrug-resistant strains in their gut microbiota [103]. Moreover, other than the microbiota, the effect of antibiotics on the neonatal health has been recently reviewed and subjected to meta-analysis by Doung et al. [104]. These authors concluded that direct exposure to antibiotics increases the risk of suffering from allergic diseases, psoriasis, atopic dermatitis, asthma, and neurodevelopment disorders. This points out to the need for understanding the impact of early life antibiotics in a wide sense, also including the gut microbiota. Actually, changes in the gut microbiota caused by antibiotic use have also been associated with an increased risk of necrotizing enterocolitis [92] and obesity [105]. Prolonged antibiotic exposure has also been associated with an increased likelihood of developing infections in the short term and obesity and inflammatory diseases in the long term, although one review did not observe significant differences depending on the time of antibiotic exposure [93]. The data on the potential long-term consequences of antibiotic exposure during infancy are further supported by animal studies assessing the long-term health effects of antibiotic-induced microbiota alteration in early life [106].

Overall, the differential effects on the gut microbiota composition have been observed after antibiotic treatment in early life, depending on factors such as type, doses, and duration of treatment. Some information about the short-term and long-lasting effects is available, but further longitudinal studies are required. 

### 5.3. Impact of Antibiotics on the Developing Infant Resistome

As previously stated, the human intestinal microbiome contains ARGs since these are found in some of the microorganisms colonizing the intestine. The infant is not an exception [107], being a potential reservoir for ARGs, even for genes to which the infant has not had exposure, such as tetracyclines or chloramphenicol [44,108]. As previously stated, infants and children constitute the human population most frequently exposed to antibiotics, with almost half of the infants having received antibiotics by the age of one year [109] and penicillin being the most commonly prescribed antibiotic [78]. During the first year of life, the number of ARGs in the individual microbiota has been reported to be above 20 genes, with some of them decreasing in occurrence with time, while the incidence of others increases [110]. The most prevalent ARGs during the first months of life are associated with resistance to erythromycin, tetracycline, aminoglycosides, and beta-lactams [111]. Unfortunately, while determining the impact of early life antibiotics on the intestinal microbiota composition has been the aim of several studies, the information of their impact upon the possession of ARGs is scarcer. In a recent systematic review, Lebeaux and co-workers [3] assessed the effects of antibiotic exposure on the gut resistome of children. The authors concluded that, although the evidence available is still limited and more research is needed, antibiotic exposure is associated with changes in ARG load. Interestingly, antibiotics administration in childhood has been repeatedly reported to increase not just the levels of genes that confer resistance to the administered antibiotic, but also of other ARGs, as shown by the increased diversity of resistance genes following antibiotic administration, although different effects seem to exist among the different antibiotics used [3,44]. A study conducted recently found that the presence of different ARGs in the intestinal microbiota of children varied after antibiotic treatment, in spite of not observing significant differences in microbial communities, indicating that antibiotic-selective pressure may have effects by strain selection, beyond changes in other taxonomic levels [41]. 

A specific, but common, form of early exposure to antibiotics is the case of intrapartum antimicrobial prophylaxis (IAP). Pregnancy has been associated with a high incidence of invasive *Streptococcus* group B (GBS) disease, which is related to a higher risk of premature delivery. GBS remains the most common culture-confirmed neonatal bacterial infection and is a significant source of neonatal morbidity globally. This procedure consists of the intrapartum administration of an antibiotic, most commonly a penicillin, to the mother to prevent the transmission of GBS to the infant during delivery. IAP has reduced the incidence of early onset neonatal disease without a notable impact on the incidence of late-onset neonatal disease [112]. However, this common practice, present in more than 30% of deliveries, has been reported to disrupt the initial establishment of the gut microbiota in both pre-term and full-term babies [113,114]. Studies that have analyzed the effect of prenatal antibiotic administration have shown that the microbiota of infants whose mothers have undergone an antibiotic treatment differs from those who have not been exposed to any antibiotics. Despite the differences found at the taxonomic level between different studies, the vast majority of reports agree that IAP decreases both the diversity and abundance of members of the *Bifidobacteriaceae* and *Bacteroidaceae* families. This practice also leads to a substantial increase in *Enterobacteriaceae* in the neonatal gut microbiota during the first weeks of life. The changes caused by IAP at the taxonomic level are comparable to those triggered by the direct administration of antibiotics to infants [115]. IAP has also been reported to cause changes in the load of ARGs in the gut microbiota of newborns. A study comparing the presence of selected ARGs in one-month-old infants found that the prevalence of *bla*TEM, *bla*CTX-M, and *aac*(6′)-*aph*(2″) was higher in fecal samples from children whose mothers had been treated with IAP [114].

Prenatal exposure to antibiotics may also be present, although whether or not this affects the evolution of ARGs in the newborn still needs to be studied. UTIs are frequently encountered in pregnant women and are easily treated with antibiotic therapy, tailored based on organism sensitivities when available from urine culture results. Short-course antibiotics commonly used include amoxicillin, ampicillin, cephalosporins, nitrofurantoin, and trimethoprim-sulfamethoxazole [116]. These common infections during pregnancy and the usual treatment of antibiotics suggest the wide exposure of pregnant women and, therefore, of the fetus, to these antibiotics. Unfortunately, the consequences for the gut microbiome and the development of the resistome of the mother and, therefore, potentially also of the baby still have to be elucidated.

It is important to underline that some covariates frequently found in infant cohorts may also have a strong impact on the possession of ARGs. For instance, antibiotic exposure during the first year of life has been associated with increases in the mupirocin resistance gene *ile*S and the beta-lactamase gene cfxA6; in infants staying at home and not attending a day-care center, only *erm*F was enriched. In day-care-attending infants, several ARGs were affected, including *mdt*N, *tet*M, *erm*A, or *acr*B [117]. However, it is important to point out that the number of studies is still limited; different antibiotics and doses are used among studies, as well as different specific uses (therapeutic vs. prophylactic). In addition, the infant microbiome is in continuous evolution during the first years of life and, thus, changes in composition will occur along time, even in healthy and unexposed infants, making the comparison of studies intervening at different infant ages difficult. Moreover, most studies to date have focused on pre-term infants, often highly exposed to antibiotics, demonstrating the long-term effects of antibiotic treatments on the resistome [44], but detailed information is not available for full-term babies.

## 6. Impact of Increased Levels of ARGs on Health and Mitigation Strategies

The most threatening problem of antibiotic-resistant microorganisms in public health is that ARGs may be acquired by pathogenic strains through horizontal transfer. Several in vitro and in vivo experiments have shown the spread of ARGs between members of the gut microbiota, such as enterobacteria or enterococci bacteria [118]. Enterobacteria are a minority population in the gut microbiome of healthy adults; however, it is present in higher levels in early life and infanthood and also in adults with gut inflammatory diseases, engaging HGT [119]. In addition, the gut microbiota in infants is immature and highly dynamic, so the risk of spreading both ARGs and antibiotic-resistant pathogens could be high. The transfer of ARGs in the infant gut has already been observed. An ampicillin resistance gene between two *E. coli* strains via plasmid transference was detected in the gut microbiota of infants treated with antibiotics [120]. Microorganisms showing resistance to antibiotics may compromise the immune system with a loss of effect in antibiotic treatments to fight routine infectious diseases, such as nosocomial and secondary infections, but also undermine the treatment of infectious complications in patients with other diseases, such as organ transplants, cancer therapies, and the treatment of chronic diseases, including asthma or diabetes [121]. This could be more accentuated in the pediatric population. To put this threat into perspective, it was estimated that resistance will cause around 300 million premature deaths by the year 2050 [122]. 

Despite the strong antimicrobial-selective pressure that can be expected after antibiotic use [47,94], the infant gut microbiota and resistome can be affected by different factors, such as the vertical transmission and perinatal factors mentioned above; it can also be due to contact with a pet or the use of antibiotics in agriculture and farms, thus affecting the ARGs levels [118,123]. An increment in ARGs in commensal bacteroides in the gut microbiota of healthy people in recent years was observed, but information in infants is not yet available. On the other hand, indirect factors can also noticeably shape the infant gut resistome and can be used as a tool for controlling and reducing antibiotic-resistant microorganisms in the gut microbiota.

Diet is known to be the main gut microbiota modulator and could also, therefore, beneficially modulate the resistome. Few studies have analyzed the effect of diet on ARGs. In one of the first studies published, breastfeeding for 6 months or longer reduced the relative abundance of ARGs in the infant microbiome, correlating with the decrement in Gammaproteobacteria and conversely an increment in bifidobacteria [45]. Interestingly, a dietary intervention based on whole-grain food, traditional Chinese medicinal foods, and prebiotics entailed a decrease in the gut resistome ARG load in obese children [124]. Probiotics could also affect the ARG abundance by the modulation of the gut microbiota. Some probiotic interventions on premature infants showed a decrease in ARG load or the amelioration of the harmful effects of antibiotics on the intestinal microbiota, leading to the possession of a resistome more similar to full-term infants [125,126]. However, in adults, opposing person-specific and antibiotic-dependent effects of probiotics on ARG burden were observed, which were related to the permissiveness to probiotic colonization [127]. The analysis of fourteen clinical studies regarding the efficacy of probiotics in the eradication of the intestinal possession of multidrug-resistant organisms (MDROs) concluded no significant effect of probiotics with respect to the placebo [128]. Further studies will be necessary to understand the role of probiotics on the infant resistome. Bacteriophages are therapeutic tools with promising in vitro results targeting gut resistome [129]. In two successful clinical studies, two resistant *K. pneumoniae* were eradicated in adult patients after a specific bacteriophage treatment [130,131]. Fecal microbiota transplantation (FMT) for antibiotic-resistant bacteria decolonization or gut microbiota modulation is another interventional tool studied in recent years. Although this intervention is a well-known treatment for patients with *Clostridium difficile*-recurrent infections, the interest in their effectiveness in reducing ARG load and antimicrobial-resistant bacteria in the gut increased based on the positive results in the above class of patients [132] and in the higher likelihood of colonization and consequent reduction in the pathogens’ colonization, compared with most common probiotics. Several cohort studies and case reports were conducted and some randomized studies evaluated the capability of FMT as a treatment for MDROs, which are currently ongoing [129,133]. Among these cohort studies, only one was conducted in pediatric immunocompromised patients scheduled to undergo allogeneic hematopoietic stem cell transplantation and having a history of systemic infections with MDROs [134].

At present, a key challenge is to control worldwide AMR dissemination and different strategies are needed, in addition to the implementation of global policies and the discovery of new drugs to restore the capacities of antibiotics. A very promising approach has emerged with a focus on the gut microbiota modulation through functional foods. More research is need to select the biological tools with a specific and more customized mode of action. In vitro studies have already highlighted the capability of probiotics, prebiotics, and even bacteriophages to control the blooming of ARG-enriched taxa, such as enterobacteria. Further steps to identify the taxonomic basis for a given shift in ARG abundance and possession would be of great value.

## Figures and Tables

**Table 1:** Most common pediatric infections and initial empiric antibiotic therapy (until culture/antibiogram is received).

NEONATAL PERIOD		
	Most Frequent Bacteria	Usual Antibiotic Treatment
Vertical infections	*Streptococcus agalactiae, Listeria monocytogenes, Enterococcus faecalis, Staphylococcus aureus, Escherichia coli*	Ampicillin IV plus Gentamicin IV
Nosocomial infections	*Staphylococcus epidermidis, coagulase negative Staphylococcus, Staphylococcus aureus, Klebsiella pneumoniae, Pseudomonas aeruginosa, Serratia marcescens, Enterobacter* spp.	Vancomycin IV plus Amikacin IV
Vertical or Nosocomial with meningitis	Any of the previous	Cefotaxime IV plus Gentamicin IV
Community infections:	*Chlamydia trachomatis, Streptococcus pneumonia,*	Oral azithromycin or
- Respiratory	*Bordetella pertussis*	Ampicillin IV
- Urinary	*E. coli, Enterococcus faecalis*	Ampicillin IV plus Gentamicin IV
- Soft tissues	*Staphylococcus aureus*	Cloxacillin IV
**TODDLER–CHILD PERIOD**		
- Otitis/sinusitis - Tonsillopharyngitis	Streptococcus pneumoniae *Haemophilus influenzae* *Streptococcus pyogenes*	Amoxicillin Amoxicillin–clavulanate Penicillin
- Pneumonia (typical) - Pneumonia (atypical)	*Streptococcus pneumoniae* *Haemophilus influenzae* *Mycoplasma pneumoniae* *Bordetella pertussis*	Amoxicillin Amoxicillin clavulanic Macrolides: azithromycin or clarithromycin
Urinary infections	*E. coli, Enterococcus faecalis, Proteus*	Amoxicillin–clavulanic, Cefixime, Cefuroxime, Cefotaxime, Ceftriaxone
Other infections - Osteomuscular - Meningitis and sepsis	*Staphylococcus aureus, Kingella kingae* *Streptococcus pneumoniae, Neisseria meningitidis,* *Haemophilus influenzae*	Cloxacillin, Cefotaxime Cefotaxime

**Table 2:** Guidelines and doses of the antibiotic therapies most frequently used in pediatrics. Doses and time depend on the gestational age and weight of neonates and the chronological age and weight of older children.

	Antibiotic	Doses (mg/kg)	Time
Neonate	Ampicillin (IV)	25–50	Every 6–8–12 h
	Gentamicin (IV)	4–5	Every 24–48 h
	Vancomycin (IV)	10–15	Every 8–12 h
	Teicoplamin (IV)	10	Every 12–24 h
	Amikacin (IV)	15–18	Every 24–48 h
	Cefotaxime (IV)	50–100	Every 8–12 h
	Cloxacillin (IV)	25–30	Every 6–12 h
Toddler–Child	Phenoxymethylpenicillin (oral)	6–12	Every 6–8 h
	Benzylpenicillin (IV IM)	25,000–50,000 *	Every 6 h
	Amoxicillin (oral)	15–25	Every 8 h
	Amoxicilin clavulanic (oral or IV)	15–25 **	Every 8 h
	Ampicillin (IV)	25–50	Every 6 h
	Cloxacillin (IV or oral)	25	Every 6 h
	Cefixime (oral)	4–8	Every 12–24 h
	Cefuroxime (oral)	10	Every 12 h
	Cefuroxime (IV)	33	Every 8 h
	Cefotaxime (IV)	25–50	Every 6–8 h
	Ceftriaxone (IM or IV)	50–100	Every 12–24 h
	Gentamicin (IV)	5	Every 24 h
	Azithromycin (oral)	10	Every 24 h
	Clarithromycin (oral)	7.5–15	Every 12 h

* UI. ** Amoxicillin doses.

## Data Availability

No new data were created or analyzed in this study. Data sharing is not applicable to this article.

## References

[1] Milani C., Duranti S., Bottacini F., Casey E., Turroni F., Mahony J., Belzer C., Delgado Palacio S., Arboleya Montes S., Mancabelli L. (2017). The First Microbial Colonizers of the Human Gut: Composition, Activities, and Health Implications of the Infant Gut Microbiota. Microbiol. Mol. Biol. Rev..

[2] Martin R., Nauta A., Ben Amor K., Knippels L., Knol J., Garssen J. (2010). Early life: Gut microbiota and immune development in infancy. Benef. Microbes..

[3] Lebeaux R.M., Karalis D.B., Lee J., Whitehouse H.C., Madan J.C., Karagas M.R., Hoen A.G. (2022). The association between early life antibiotic exposure and the gut resistome of young children: A systematic review. Gut Microbes.

[4] Kounnavong S., Yan W., Sihavong A., Sychareun V., Eriksen J., Hanson C., Chaleunvong K., Keohavong B., Vongsouvath M., Mayxay M. (2022). Antibiotic knowledge, attitudes and reported practice during pregnancy and six months after birth: A follow- up study in Lao PDR. BMC Pregnancy Childbirth.

[5] Cantey J.B., Wozniak P.S., Sanchez P.J. (2015). Prospective surveillance of antibiotic use in the neonatal intensive care unit: Results from the SCOUT study. Pediatr. Infect. Dis. J..

[6] Dekker A.R.J., Verheij T.J.M., van der Velden A.W. (2017). Antibiotic management of children with infectious diseases in Dutch primary care. Fam. Pract..

[7] Taine M., Offredo L., Dray-Spira R., Weill A., Chalumeau M., Zureik M. (2021). Paediatric outpatient prescriptions in France between 2010 and 2019: A nationwide population-based study: Paediatric outpatient prescriptions in France, 2010 to 2019. Lancet Reg Health Eur..

[8] Baron R., Taye M., der Vaart I.B., Ujcic-Voortman J., Szajewska H., Seidell J.C., Verhoeff A. (2020). The relationship of prenatal antibiotic exposure and infant antibiotic administration with childhood allergies: A systematic review. BMC Pediatr..

[9] Li G., Bielicki J.A., Ahmed A.S.M.N.U., Islam M.S., Berezin E.N., Gallacci C.B., Guinsburg R., da Silva Figueiredo C.E., Santarone Vieira R., Silva A.R. (2020). Towards understanding global patterns of antimicrobial use and resistance in neonatal sepsis: Insights from the NeoAMR network. Arch. Dis. Child..

[10] (2021). WHO. https://www.who.int/news-room/fact-sheets/detail/antimicrobial-resistance.

[11] Antimicrobial Resistance Collaborators (2022). Global burden of bacterial antimicrobial resistance in 2019: A systematic analysis. Lancet.

[12] Antimicrobial Resistance in the EU/EEA: A One Health Response. OECD 2022. https://www.ecdc.europa.eu/sites/default/files/documents/antimicrobial-resistance-policy-brief-2022.pdf.

[13] Roope L.S.J., Smith R.D., Pouwels K.B., Buchanan J., Abel L., Eibich P., Butler C.C., Tan P.S., Walker A.S., Robotham J.V. (2019). The challenge of antimicrobial resistance: What economics can contribute. Science.

[14] Bartoloni A., Pallecchi L., Rodríguez H., Fernandez C., Mantella A., Bartalesi F., Strohmeyer M., Kristiansson C., Gotuzzo E., Paradisi F. (2009). Antibiotic resistance in a very remote Amazonas community. Int. J. Antimicrob. Agents.

[15] Hollis A., Ahmed Z. (2014). The path of least resistance: Paying for antibiotics in non-human uses. Health Policy.

[16] Browne A.J., Chipeta M.G., Haines-Woodhouse G., Kumaran E.P.A., Hamadani B.H.K., Zaraa S., Henry N.J., Deshpande A., Reiner R.C., Day N.P.J. (2021). Global antibiotic consumption and usage in humans, 2000–2018: A spatial modelling study. Lancet Plan Health.

[17] Rahman S., Kesselheim A.S., Hollis A. (2023). Persistence of resistance: A panel data analysis of the effect of antibiotic usage on the prevalence of resistance. J. Antibiot..

[18] Kim D.W., Cha C.J. (2021). Antibiotic resistome from the One-Health perspective: Understanding and controlling antimicrobial resistance transmission. Exp. Mol. Med..

[19] Jorgensen J.H. (1997). Laboratory issues in the detection and reporting of antibacterial resistance. Infect. Dis. Clin. N. Am..

[20] Lu N., Hu Y., Zhu L., Yang X., Yin Y., Lei F., Zhu Y., Du Q., Wang X., Meng Z. (2014). DNA microarray analysis reveals that antibiotic resistance-gene diversity in human gut microbiota is age related. Sci. Rep..

[21] Whon T.W., Shin N.R., Kim J.Y., Roh S.W. (2021). Omics in gut microbiome analysis. J. Microbiol..

[22] Finegold S.M. (1970). Interaction of antimicrobial therapy and intestinal flora. Am. J. Clin. Nutr..

[23] Archer G.L., Penell E. (1990). Detection of methicillin resistance in Staphylococci by using a DNA probe. Antimicrob. Agents Chemother..

[24] Gosalbes M.J., Vallès Y., Jiménez-Hernández N., Balle C., Riva P., Miravet-Verde S., de Vries L.E., Llop S., Agersø Y., Sørensen S.J. (2016). High frequencies of antibiotic resistance genes in infants’ meconium and early fecal samples. J. Dev. Orig. Health Dis..

[25] Klassert T.E., Zubiria-Barrera C., Kankel S., Stock M., Neubert R., Lorenzo-Diaz F., Doehring N., Driesch D., Fischer D., Slevogt H. (2020). Early bacterial colonization and antibiotic resistance gene acquisition in newborns. Front. Cell. Infect. Microbiol..

[26] Lai F.Y., Muziasari W., Virta M., Wiberg K., Ahrens L. (2021). Profiles of environmental antibiotic resistomes in the urban aquatic recipients of Sweden using high-throughput quantitative PCR analysis. Environ. Pol..

[27] Pehrsson E.C., Forsberg K.J., Gibson M.K., Ahmadi S., Dantas G. (2013). Novel resistance functions uncovered using functional metagenomic investigations of resistance reservoirs. Front. Microbiol..

[28] Kim D.W., Thawng C.N., Choi J.H., Lee K., Cha C.J. (2018). Polymorphism of antibiotic-inactivating enzyme driven by ecology expands the environmental resistome. ISME J..

[29] Böhm M.E., Razavi M., Marathe N.P., Flach C.F., Larsson D.G.J. (2020). Discovery of a novel integron-borne aminoglycoside resistance gene present in clinical pathogens by screening environmental bacterial communities. Microbiome.

[30] Ma L., Xia Y., Li B., Yang Y., Li L.G., Tiedje J.M., Zhang G. (2016). Metagenomic assembly reveals hosts of antibiotic resistance genes and the shared resistome in pig, chicken, and human feces. Environ. Sci. Technol..

[31] Suzuki S., Horinouchi T., Furusawa C. (2014). Prediction of antibiotic resistance by gene expression profiles. Nat. Commun..

[32] Felden B., Cattoir V. (2018). Bacterial adaptation to antibiotics through regulatory RNAs. Antimicrob. Agents Chemother..

[33] Le Neindre K., Dejoies L., Reissier S., Guérin F., Felden B., Cattoir V. (2022). Small RNA-mediated regulation of the tet(M) resistance gene expression in Enterococcus faecium. Res. Microbiol..

[34] Boolchandani M., D’Souza A.W., Dantas G. (2019). Sequencing-based methods and resources to study antimicrobial resistance. Nat. Rev. Genet..

[35] Davis J.J., Boisvert S., Brettin T., Kenyon R.W., Mao C., Olson R., Overbeek R., Santerre J., Shukla M., Wattam A.R. (2016). Antimicrobial resistance prediction in PATRIC and RAST. Sci. Rep..

[36] Arango-Argoty G.A., Dai D., Pruden A., Vikesland P., Heath L.S., Zhang L. (2019). NanoARG: A web service for detecting and contextualizing antimicrobial resistance genes from nanopore-derived metagenomes. Microbiome.

[37] Bokulich N.A., Chung J., Battaglia T., Henderson N., Jay M., Li H., Lieber A., Wu F., Perez-Perez G.I., Chen Y. (2016). Antibiotics, birth mode, and diet shape microbiome maturation during early life. Sci. Transl. Med..

[38] Penders J., Stobberingh E.E., Savelkoul P.H., Wolffs P.F. (2013). The human microbiome as a reservoir of antimicrobial resistance. Front. Microbiol..

[39] Leo S., Curtis N., Zimmermann P. (2022). The neonatal intestinal resistome and factors that influence it-a systematic review. Clin. Microbiol. Infect..

[40] Thänert R., Sawhney S.S., Schwartz D.J., Dantas G. (2022). The resistance within: Antibiotic disruption of the gut microbiome and resistome dynamics in infancy. Cell Host Microbe.

[41] Li X., Stokholm J., Brejnrod A., Vestergaard G.A., Russel J., Trivedi U., Thorsen J., Gupta S., Hjelmsø M.H., Shah S.A. (2021). The infant gut resistome associates with E. coli, environmental exposures, gut microbiome maturity, and asthma-associated bacterial composition. Cell Host Microbe.

[42] Reyman M., van Houten M.A., van Baarle D., Bosch A.A.T.M., Man W.H., Chu M.L.J.N., Arp K., Watson R.L., Sanders E.A.M., Fuentes S. (2019). Impact of delivery mode-associated gut microbiota dynamics on health in the first year of life. Nat. Commun..

[43] Li W., Tapiainen T., Brinkac L., Lorenzi H.A., Moncera K., Tejesvi M.V., Salo J., Nelson K.E. (2021). Vertical transmisión of gut microbiome and antimicrobial resistance genes in infants exposed to antibiotics at birth. J. Infect. Dis..

[44] Gasparrini A.J., Wang B., Sun X., Kennedy E.A., Hernandez-Leyva A., Ndao I.M., Tarr P.I., Warner B.B., Dantas G.l. (2019). Persistent metagenomic signatures of early-life hospitalization and antibiotic treatment in the infant gut microbiota and resistome. Nat. Microbiol..

[45] Pärnänen K., Karkman A., Hultman J., Lyra C., Bengtsson-Palme J., Larsson D.G.J., Rautava S., Isolauri E., Salminen S., Kumar H. (2018). Maternal gut and breast milk microbiota affect infant gut antibiotic resistome and mobile genetic elements. Nat. Commun..

[46] Socha-Banasiak A., Pawłowska M., Czkwianianc E., Pierzynowska K. (2021). From Intrauterine to Extrauterine Life-The Role of Endogenous and Exogenous Factors in the Regulation of the Intestinal Microbiota Community and Gut Maturation in Early Life. Front. Nutr..

[47] Gibson M.K., Wang B., Ahmadi S., Burnham C.A., Tarr P.I., Warner B.B., Dantas G. (2016). Developmental dynamics of the preterm infant gut microbiota and antibiotic resistome. Nat. Microbiol..

[48] Zhuang L., Chen H., Zhang S., Zhuang J., Li Q., Feng Z. (2019). Intestinal Microbiota in Early Life and Its Implications on Childhood Health. Genom. Proteom. Bioinform..

[49] Hill C.J., Lynch D.B., Murphy K., Ulaszewska M., Jeffery I.B., O'Shea C.A., Watkins C., Dempsey E., Mattivi F., Tuohy K. (2017). Evolution of gut microbiota composition from birth to 24 weeks in the INFANTMET Cohort. Microbiome.

[50] Mesa M.D., Loureiro B., Iglesia I., Fernandez Gonzalez S., Llurba Olivé E., García Algar O., Solana M.J., Cabero Perez M.J., Sainz T., Martinez L. (2020). The Evolving Microbiome from Pregnancy to Early Infancy: A Comprehensive Review. Nutrients.

[51] Neuman H., Koren O. (2017). The Pregnancy Microbiome. Nestlé Nutr. Inst. Work. Ser..

[52] Khan I., Yasir M., Farman M., Kumosani T., AlBasri S.F., Bajouh O.S., Azhar E.I. (2019). Evaluation of gut bacterial community composition and antimicrobial resistome in pregnant and non-pregnant women from Saudi population. Infect. Drug Resist..

[53] Sosa-Moreno A., Comstock S.S., Sugino K.Y., Ma T.F., Paneth N., Davis Y., Olivero R., Schein R., Maurer J., Zhang L. (2020). Perinatal risk factors for fecal antibiotic resistance gene patterns in pregnant women and their infants. PLoS ONE.

[54] Gupta P., Singh M.P., Goyal K. (2020). Diversity of Vaginal Microbiome in Pregnancy: Deciphering the Obscurity. Front. Public Health.

[55] Severgnini M., Camboni T., Ceccarani C., Morselli S., Cantiani A., Zagonari S., Patuelli G., Pedna M.F., Sambri V., Foschi C. (2021). Distribution of ermb, ermf, tet(W), and tet(m) resistance genes in the vaginal ecosystem of women during pregnancy and puerperium. Pathogens.

[56] Maestre-Carballa L., Navarro-López V., Martinez-Garcia M.A. (2022). Resistome Roadmap: From the Human Body to Pristine Environments. Front. Microbiol..

[57] Roachford O.S.E., Alleyne A.T., Kuelbs C., Torralba M.G., Nelson K.E. (2021). The cervicovaginal microbiome and its resistome in a random selection of Afro-Caribbean women. Human. Microb. J..

[58] Perera M., Al-Hebshi N.N., Speicher D.J., Perera I., Johnson N.W. (2016). Emerging role of bacteria in oral carcinogenesis: A review with special reference to perio-pathogenic bacteria. J. Oral. Microbiol..

[59] Borgo P.V., Rodrigues V.A.A., Feitosa A.C.R., Xavier K.C.B., Avila-Campos M.J. (2014). Association between periodontal condition and subgingival microbiota in women during pregnancy: A longitudinal study. J. Appl. Oral. Sci..

[60] Fujiwara N., Tsuruda K., Iwamoto Y., Kato F., Odaki T., Yamane N., Hori Y., Harashima Y., Sakoda A., Tagaya A. (2017). Significant increase of oral bacteria in the early pregnancy period in Japanese women. J. Investig. Clin. Dent..

[61] Gohir W., Kennedy K.M., Wallace J.G., Saoi M., Bellissimo C.J., Britz-McKibbin P., Petrik J.J., Surette M.G., Sloboda D.M. (2019). High-fat diet intake modulates maternal intestinal adaptations to pregnancy and results in placental hypoxia, as well as altered fetal gut barrier proteins and immune markers. J. Physiol..

[62] Serrano M.G., Parikh H.I., Brooks J.P., Edwards D.J., Arodz T.J., Edupuganti L., Huang B., Girerd P.H., Bokhari Y.A., Bradley S.P.- (2019). Racioethnic diversity in the dynamics of the vaginal microbiome during pregnancy. Nat. Med..

[63] Carvalho M.J., Sands K., Thomson K., Portal E., Mathias J., Milton R., Gillespie D., Dyer C., Akpulu C., Boostrom I. (2022). Antibiotic resistance genes in the gut microbiota of mothers and linked neonates with or without sepsis from low- and middle-income countries. Nat. Microbiol..

[64] Yassour M., Jason E., Hogstrom L.J., Arthur T.D., Tripathi S., Siljander H., Selvenius J., Oikarinen S., Hyöty H., Virtanen S.M. (2018). Strain-level analysis of mother-to-child bacterial transmission during the first few months of life. Cell Host Microbe.

[65] Kozak K., Charbonneau D., Sanozky-Dawes R., Klaenhammer T. (2015). Characterization of bacterial isolates from the microbiota of mothers’ breast milk and their infants. Gut Microbes.

[66] Patangia D.V., Ryan C.A., Dempsey E., Stanton C., Ross R.P. (2022). Vertical transfer of antibiotics and antibiotic resistant strains across the mother/baby axis. Trends Microbiol..

[67] Dubois V., De Barbeyrac B., Rogues A.M., Arpin C., Coulange L., Andre C., M'zali F., Megraud F., Quentin C. (2010). CTX-M-producing Escherichia coli in a maternity ward: A likely community importation and evidence of mother-to-neonate transmission. J. Antimicrob. Chemother..

[68] Alicea-Serrano A.M., Contreras M., Magris M., Hidalgo G., Dominguez-Bello M.G. (2013). Tetracycline resistance genes acquired at birth. Arch. Microbiol..

[69] Karami N., Nowrouzian F., Adlerberth I., Wold A.E. (2006). Tetracycline resistance in Escherichia coli and persistence in the infantile colonic microbiota. Antimicrob. Agents Chemother..

[70] Das L., Virmani R., Sharma V., Rawat D., Singh Y. (2019). Human Milk Microbiota: Transferring the Antibiotic Resistome to Infants. Ind. J. Microbiol..

[71] Huang M.S., Cheng C.C., Tseng S.Y., Lin Y.L., min Lo H., Chen P.W. (2019). Most commensally bacterial strains in human milk of healthy mothers display multiple antibiotic resistance. Microbiologyopen.

[72] Nadimpalli M.L., Bourke C.D., Robertson R.C., Delarocque-Astagneau E., Manges A.R., Pickering A.J. (2020). Can breastfeeding protect against antimicrobial resistance?. BMC Med..

[73] Li X., Zhou Y., Zhan X., Huang W., Wang X. (2018). Breast milk is a potential reservoir for livestock-associated Staphylococcus aureus and community-associated Staphylococcus aureus in Shanghai, China. Front. Microbiol..

[74] Chen P.W., Tseng S.Y., Huang M.S. (2016). Antibiotic Susceptibility of Commensal Bacteria from Human Milk. Curr. Microbiol..

[75] White N.D. (2022). Drug-Induced Microbiome Changes: Considerations in Pregnancy. Am. J. Lifestyle Med..

[76] Brigadoi G., Rossin S., Visentin D., Barbieri E., Giaquinto C., Da Dalt L., Donà D. (2023). The impact of Antimicrobial Stewardship Programmes in paediatric emergency departments and primary care: A systematic review. Ther. Adv. Infect. Dis..

[77] Calle-Miguel L., Pérez-Méndez C., García-García E., Moreno-Pavón B., Solís-Sánchez G. (2022). Trends and Pattern of Antibiotic Use in Children in Northern Spain, Interpreting Data about Antibiotic Consumption in Pediatric Outpatients. Children.

[78] Poole N.M., Shapiro D.J., Fleming-Dutra K.E., Hicks L.A., Hersh A.L., Kronman M.P. (2019). Antibiotic Prescribing for Children in United States Emergency Departments: 2009–2014. Pediatrics.

[79] García-Moreno F.J., Escobar-Castellanos M., Marañón R., Rivas-García A., Manrique-Rodríguez S., Mora-Capín A., Fernández-Llamazares C.M. (2022). Adequacy of pediatric antimicrobial prescribing in the Emergency Department at discharge. An. Pediatr. (Engl. Ed.).

[80] WHO (2022). Collaborating Center for Drug Statistics Methodology. Guidelines for ATC Classification and DDD Assignment.

[81] Same R.G. (2022). The Current State and Future Directions of Inpatient Pediatric Antimicrobial Stewardship. Infect. Dis. Clin. N. Am..

[82] Glaser M.A., Hughes L.M., Jnah A., Newberry D. (2021). Neonatal Sepsis: A Review of Pathophysiology and Current Management Strategies. Adv. Neonatal Care.

[83] Fleiss N., Schwabenbauer K., Randis T.M., Polin R.A. (2023). What's new in the management of neonatal early-onset sepsis?. Arch. Dis. Child. Fetal Neonatal Ed..

[84] Cailes B., Kortsalioudaki C., Buttery J., Pattnayak S., Greenough A., Matthes J., Bedford Russell A., Kennea N., Heath P.T., neonIN network (2018). Epidemiology of UK neonatal infections: The neonIN infection surveillance network. Arch. Dis. Child. Fetal Neonatal Ed..

[85] Paul S.P., Khattak H., Kini P.K., Heaton P.A., Goel N. (2022). NICE guideline review: Neonatal infection: Antibiotics for prevention and treatment (NG195). Arch. Dis. Child. Educ. Pract. Ed..

[86] Winzor G., Atabani S.F. (2022). How and when to use CSF to investigate neonates and children with possible central nervous system infection. Arch. Dis. Child. Educ. Pract. Ed..

[87] Siddiq S., Grainger J. (2015). The diagnosis and management of acute otitis media: American Academy of Pediatrics Guidelines 2013. Arch. Dis. Child. Educ. Pract. Ed..

[88] Kim N.N., Marikar D. (2020). Antibiotic prescribing for upper respiratory tract infections: NICE guidelines. Arch. Dis. Child. Educ. Pract. Ed..

[89] Autore G., Bernardi L., La Scola C., Ghidini F., Marchetti F., Pasini A., Pierantoni L., Castellini C., Gatti C., Malaventura C. (2022). The Uti-Ped-Er Study Group. Management of Pediatric Urinary Tract Infections: A Delphi Study. Antibiotics.

[90] Yi J., Wood J.B., Creech C.B., Williams D., Jimenez-Truque N., Yildirim I., Sederdahl B., Daugherty M., Hussaini L., Munye M. (2021). Clinical Epidemiology and Outcomes of Pediatric Musculoskeletal Infections. J. Pediatr..

[91] Posadas E., Fisher J. (2018). Pediatric bacterial meningitis: An update on early identification and management. Pediatr. Emer. Med. Pract..

[92] Zimmermann P., Curtis N. (2019). The effect of antibiotics on the composition of the intestinal microbiota—A systematic review. J. Infect..

[93] Fjalstad J.W., Esaiassen E., Juvet L.K., van den Anker J.N., Klingenberg C. (2018). Antibiotic therapy in neonates and impact on gut microbiota and antibiotic resistance development: A systematic review. J. Antimicrob. Chemother..

[94] Yassour M., Vatanen T., Siljander H., Hämäläinen A.M., Härkönen T., Ryhänen S.J., Franzosa E.A., Vlamakis H., Huttenhower C., Gevers D. (2016). Natural history of the infant gut microbiome and impact of antibiotic treatment on bacterial strain diversity and stability. Sci. Transl. Med..

[95] Fouhy F., Guinane C.M., Hussey S., Wall R., Ryan C.A., Dempsey E.M., Murphy B., Ross R.P., Fitzgerald G.F., Stanton C. (2012). High-throughput sequencing reveals the incomplete, short-term recovery of infant gut microbiota following parenteral antibiotic treatment with ampicillin and gentamicin. Antimicrob. Agents Chemother..

[96] Uzan-Yulzari A., Turta O., Belogolovski A., Ziv O., Kunz C., Perschbacher S., Neuman H., Pasolli E., Oz A., Ben-Amram H. (2021). Neonatal antibiotic exposure impairs child growth during the first six years of life by perturbing intestinal microbial colonization. Nat. Commun..

[97] Reyman M., van Houten M.A., Watson R.L., Chu M.L.J.N., Arp K., de Waal W.J., Schiering I., Plötz F.B., Willems R.J.L., van Schaik W. (2022). Effects of early-life antibiotics on the developing infant gut microbiome and resistome: A randomized trial. Nat. Commun..

[98] Gibson M.K., Crofts T.S., Dantas G. (2015). Antibiotics and the developing infant gut microbiota and resistome. Curr. Opin. Microbiol..

[99] Zwittink R.D., Renes I.B., van Lingen R.A., van Zoeren-Grobben D., Konstanti P., Norbruis O.F., Martin R., Groot Jebbink L.J.M., Knol J., Belzer C. (2018). Association between duration of intravenous antibiotic administration and early-life microbiota development in late-preterm infants. Eur. J. Clin. Microbiol. Infect. Dis..

[100] Kuppala V.S., Meinzen-Derr J., Morrow A.L., Schibler K.R. (2011). Prolonged initial empirical antibiotic treatment is associated with adverse outcomes in premature infants. J. Pediatr..

[101] Greenwood C., Morrow A.L., Lagomarcino A.J., Altaye M., Taft D.H., Yu Z., Newburg D.S., Ward D.V., Schibler K.R. (2014). Early empiric antibiotic use in preterm infants is associated with lower bacterial diversity and higher relative abundance of Enterobacter. J. Pediatr..

[102] Rooney A.M., Timberlake K., Brown K.A., Bansal S., Tomlinson C., Lee K.S., Science M., Coburn B. (2020). Each Additional Day of Antibiotics Is Associated with Lower Gut Anaerobes in Neonatal Intensive Care Unit Patients. Clin. Infect. Dis..

[103] Schwartz D.J., Langdon A.E., Dantas G. (2020). Understanding the impact of antibiotic perturbation on the human microbiome. Genome Med..

[104] Duong Q.A., Pittet L.F., Curtis N., Zimmermann P. (2022). Antibiotic exposure and adverse long-term health outcomes in children: A systematic review and meta-analysis. J. Infect..

[105] Saari A., Virta L.J., Sankilampi U., Dunkel L., Saxen H. (2015). Antibiotic exposure in infancy and risk of being overweight in the first 24 months of life. Pediatrics.

[106] Cox L.M., Yamanishi S., Sohn J., Alekseyenko A.V., Leung J.M., Cho I., Kim S.G., Li H., Gao Z., Mahana D. (2014). Altering the intestinal microbiota during a critical developmental window has lasting metabolic consequences. Cell.

[107] Samarra A., Esteban-Torres M., Cabrera-Rubio R., Bernabeu M., Arboleya S., Gueimonde M., Collado M.C. (2023). Maternal-infant antibiotic resistance genes transference: What do we know?. Gut Microbes.

[108] Gueimonde M., Salminen S., Isolauri E. (2006). Presence of specific antibiotic (tet) resistance genes in infant faecal microbiota. FEMS Immunol. Med. Microbiol..

[109] Stam J., van Stuijvenberg M., Grüber C., Mosca F., Arslanoglu S., Chirico G., Braegger C.P., Riedler J., Boehm G., Sauer P.J. (2012). Antibiotic use in infants in the first year of life in five European countries. Acta Paediatr..

[110] Loo E.X.L., Zain A., Yap G.C., Purbojati R.W., Drautz-Moses D.I., Koh Y.Q., Chong Y.S., Tan K.H., Gluckman P.D., Yap F. (2020). Longitudinal assessment of antibiotic resistance gene profiles in gut microbiomes of infants at risk of eczema. BMC Infect. Dis..

[111] Nogacka A.M., Salazar N., Arboleya S., Suárez M., Fernández N., Solís G., de Los Reyes-Gavilán C.G., Gueimonde M. (2018). Early microbiota, antibiotics and health. Cell. Mol. Life Sci..

[112] Raabe V.N., Shane A.L. (2019). Group B Streptococcus (*Streptococcus agalactiae*). Microbiol. Spect..

[113] Arboleya S., Sánchez B., Milani C., Duranti S., Solís G., Fernández N., de los Reyes-Gavilán C.G., Ventura M., Margolles A., Gueimonde M. (2015). Intestinal microbiota development in preterm neonates and effect of perinatal antibiotics. J. Pediatr..

[114] Nogacka A., Salazar N., Suárez M., Milani C., Arboleya S., Solís G., Fernández N., Alaez L., Hernández-Barranco A.M., de Los Reyes-Gavilán C.G. (2017). Impact of intrapartum antimicrobial prophylaxis upon the intestinal microbiota and the prevalence of antibiotic resistance genes in vaginally delivered full-term neonates. Microbiome.

[115] Arboleya S., Saturio S., Gueimonde M. (2022). Impact of intrapartum antibiotics on the developing microbiota: A review. Microb. Res. Rep..

[116] Gilstrap L.C., Ramin S.M. (2001). Urinary tract infections during pregnancy. Obstet. Gynecol. Clin. N. Am..

[117] Lebeaux R.M., Madan J.C., Nguyen Q.P., Coker M.O., Dade E.F., Moroishi Y., Palys T.J., Ross B.D., Pettigrew M.M., Morrison H.G. (2022). Impact of antibiotics on off-target infant gut microbiota and resistance genes in cohort studies. Pediatr. Res..

[118] Crits-Christoph A., Hallowell H.A., Koutouvalis K., Suez J. (2022). Good microbes, bad genes? The dissemination of antimicrobial resistance in the human microbiome. Gut Microbes.

[119] Stecher B., Denzler R., Maier L., Bernet F., Sanders M.J., Pickard D.J., Barthel M., Westendorf A.M., Krogfelt K.A., Walker A.W. (2012). Gut inflammation can boost horizontal gene transfer between pathogenic and commensal Enterobacteriaceae. Proc. Natl. Acad. Sci. USA.

[120] Goren M.G., Carmeli Y., Schwaber M.J., Chmelnitsky I., Schechner V., Navon-Venezia S. (2010). Transfer of carbapenem-resistant plasmid from Klebsiella pneumoniae ST258 to Escherichia coli in patient. Emerg. Infect. Dis..

[121] (2013). ARTITUS 2013. Antibiotic Resistance Threats in the United States. https://www.cdc.gov/drugresistance/pdf/ar-threats-2013-508.pdf.

[122] O’Neill J. (2016). Tackling Drug-Resistant Infections Globally: Final Report and Recommendations.

[123] Cifuentes S.G., Graham J., Loayza F., Saraiva C., Salinas L., Trueba G., Cárdenas P.A. (2022). Evaluation of changes in the faecal resistome associated with children’s exposure to domestic animals and food animal production. J. Glob. Antimicrob. Resist..

[124] Wu G., Zhang C., Wang J., Zhang F., Wang R., Shen J., Wang L., Pang X., Zhang X., Zhao L. (2016). Diminution of the gut resistome after a gut microbiota-targeted dietary intervention in obese children. Sci. Rep..

[125] Guitor A.K., Yousuf E.I., Raphenya A.R., Hutton E.K., Morrison K.M., McArthur A.G., Wright G.D., Stearns J.C. (2022). Capturing the antibiotic resistome of preterm infants reveals new benefits of probiotic supplementation. Microbiome.

[126] Bargheet A., Klingenberg C., Esaiassen E., Hjerde E., Cavanagh J.P., Bengtsson-Palme J., Pettersen V.K. (2023). Development of early life gut resistome and mobilome across gestational ages and microbiota-modifying treatments. EBioMedicine.

[127] Montassier E., Valdés-Mas R., Batard E., Zmora N., Dori-Bachash M., Suez J., Elinav E. (2021). Probiotics impact the antibiotic resistance gene reservoir along the human GI tract in a person-specific and antibiotic-dependent manner. Nat. Microbiol..

[128] Karbalaei M., Keikha M. (2022). Probiotics and intestinal decolonization of antibiotic-resistant microorganisms; A reality or fantasy?. Ann. Med. Surg..

[129] Merrick B., Sergaki C., Edwards L., Moyes D.L., Kertanegara M., Prossomariti D., Shawcross D.L., Goldenberg S.D. (2023). Modulation of the Gut Microbiota to Control Antimicrobial Resistance (AMR)—A Narrative Review with a Focus on Faecal Microbiota Transplantation (FMT). Infect. Dis. Rep..

[130] Corbellino M., Kieffer N., Kutateladze M., Balarjishvili N., Leshkasheli L., Askilashvili L., Tsertsvadze G., Rimoldi S.G., Nizharadze D., Hoyle N. (2019). Eradication of a Multidrug-Resistant, Carbapenemase-Producing Klebsiella pneumoniae Isolate Following Oral and Intra-rectal Therapy with a Custom Made, Lytic Bacteriophage Preparation. Clin. Infect. Dis..

[131] Kuipers S., Ruth M.M., Mientjes M., de Sévaux R.G.L., van Ingen J.A. (2019). Dutch Case Report of Successful Treatment of Chronic Relapsing Urinary Tract Infection with Bacteriophages in a Renal Transplant Patient. Antimicrob. Agents Chemother..

[132] Millan B., Park H., Hotte N., Mathieu O., Burguiere P., Tompkins T.A., Kao D., Madsen K.L. (2016). Fecal Microbial Transplants Reduce Antibiotic-resistant Genes in Patients with Recurrent Clostridium difficile Infection. Clin. Infect. Dis..

[133] Bilsen M.P., Lambregts M.M.C., van Prehn J., Kuijper E.J. (2022). Faecal microbiota replacement to eradicate antimicrobial resistant bacteria in the intestinal tract—A systematic review. Curr. Opin. Gastroenterol..

[134] Merli P., Putignani L., Ruggeri A., Del Chierico F., Gargiullo L., Galaverna F., Gaspari S., Pagliara D., Russo A., Pane S. (2020). Decolonization of multidrug resistant bacteria by fecal microbiota transplantation in five pediatric patients before allogeneic hematopoietic stem cell transplantation: Gut microbiota profiling, infectious and clinical outcomes. Haematologica.

